# A Community-Supported Clinic-Based Program for Prevention of Violence against Pregnant Women in Rural Kenya

**DOI:** 10.1155/2013/736926

**Published:** 2013-04-29

**Authors:** Janet M. Turan, Abigail M. Hatcher, Merab Odero, Maricianah Onono, Jannes Kodero, Patrizia Romito, Emily Mangone, Elizabeth A. Bukusi

**Affiliations:** ^1^Department of Health Care Organization and Policy, School of Public Health, University of Alabama at Birmingham, Birmingham, AL 35294, USA; ^2^University of California, San Francisco, San Francisco, CA 94105, USA; ^3^Wits Reproductive Health and HIV Institute, University of the Witwatersrand, Johannesburg 2001, South Africa; ^4^Kenya Medical Research Institute (KEMRI), P.O. Box 54840-00200, Nairobi, Kenya; ^5^Università di Trieste, 34134 Trieste, Italy

## Abstract

*Objective*. Pregnant women are especially vulnerable to adverse outcomes related to HIV infection and gender-based violence (GBV). We aimed at developing a program for prevention and mitigation of the effects of GBV among pregnant women at an antenatal clinic in rural Kenya. *Methods*. Based on formative research with pregnant women, male partners, and service providers, we developed a GBV program including comprehensive clinic training, risk assessments in the clinic, referrals supported by community volunteers, and community mobilization. To evaluate the program, we analyzed data from risk assessment forms and conducted focus groups (*n* = 2 groups) and in-depth interviews (*n* = 25) with healthcare workers and community members. *Results*. A total of 134 pregnant women were assessed during a 5-month period: 49 (37%) reported violence and of those 53% accepted referrals to local support resources. Qualitative findings suggested that the program was acceptable and feasible, as it aided pregnant women in accessing GBV services and raised awareness of GBV. Community collaboration was crucial in this low-resource setting. *Conclusion*. Integrating GBV programs into rural antenatal clinics has potential to contribute to both primary and secondary GBV prevention. Following further evaluation, this model may be deemed applicable for rural communities in Kenya and elsewhere in East Africa.

## 1. Introduction

Gender-based violence (GBV) is a major source of preventable mortality and morbidity for women globally [1–3]. In Kenya, 47% of ever-married women report having ever experienced emotional, physical, and/or sexual violence from their spouse—among the highest rates in the world [4, 5]. Violence towards pregnant women in Kenya is estimated to be 13.5% [6], a higher prevalence than many conditions routinely screened for during pregnancy [7]. Global research suggests that when pregnant women experience GBV, there is a higher likelihood of miscarriage [3, 8], premature labor [9], low birthweight [8, 10, 11], and infant death [12]. Demographic Health Survey data from Kenya suggests that experiencing lifetime GBV is associated with child stunting and under-2 mortality [12].

GBV is also a driver of the global HIV epidemic, particularly in sub-Saharan Africa where women are disproportionately at risk of both GBV and HIV infection. GBV increases risk of HIV acquisition [13, 14], and HIV-positive women are more likely to experience GBV than their HIV-negative counterparts [15]. Pregnant women are especially vulnerable to the intersecting risks and adverse outcomes related to HIV infection and GBV. There is evidence that pregnant women have a higher risk of HIV acquisition and transmission than other women [16, 17]. 

In addition to direct effects of GBV on maternal and child health, GBV may indirectly worsen health by reducing pregnant women's uptake of essential maternity and HIV services. In Kenya, women who anticipated partner stigma or violence were more than two times more likely to refuse HIV testing during antenatal care [18]. HIV-positive women who fear violence or a relationship breakup are less likely to enroll in HIV care [19] and may choose not to deliver at health facilities for fear of violence triggered by HIV testing or unwanted disclosure [20, 21]. A pregnant woman is often the first family member to be tested for HIV due to her contact with health services, putting her at disproportionate risk of suffering from HIV-related stigma and discrimination, in some cases leading to violence [22]. We have found that important triggers of GBV experienced by pregnant women in rural Kenya are testing for HIV without the husband's permission and disclosure of HIV-positive status during pregnancy [23].

Despite growing recognition of the urgent need for primary and secondary prevention of GBV, there are few best practices for integrating GBV services into primary healthcare settings in low- and middle-income countries [24]. Of the existing healthcare interventions for GBV identified in recent systematic reviews, none are located in sub-Saharan Africa [25, 26]. The knowledge base is especially limited for rural settings, as existing GBV services are generally urban and hospital-based [24, 27, 28]. 

There is an urgent need to integrate GBV programs into health services in rural areas and to link them with existing community structures and support services [24, 29]. In “systems-level integration,” basic services, such as screening and medical care, are delivered at one facility, with external referrals to other facilities for specialized services [24]. Our team developed and piloted such a systems-level integrated program at the primary-care level. The integrated program aimed at (a) offering risk assessment, medical care, and supported referrals for pregnant women experiencing violence at the clinic-level (secondary prevention) and (b) influencing social norms at the community level to prevent GBV (primary prevention). Findings from the formative research conducted to inform creation of the program are presented elsewhere [23]. Here, we describe the integrated GBV program conducted at a rural primary-care facility in Nyanza Province, Kenya, and present data for evaluation of the program.

## 2. Methods

### 2.1. Setting

Kenya is one of the sub-Saharan African countries greatly affected by HIV and AIDS [30]. Nyanza Province has the highest HIV prevalence in Kenya, with 15% of persons 15–49 years of age testing HIV positive [31]. It also has the highest reported prevalence of physical violence against women in the nation, with 57% of women in Nyanza aged 15–49 reporting having ever experienced physical violence since age 15, and 36% of these women reporting physical violence in the past 12 months [4]. Sixty percent of ever-married women aged 15–49 in Nyanza report ever having experienced emotional, physical, or sexual violence committed by their husband partner (39% reported emotional, 51% physical, and 22% sexual) [32]. This study was conducted in collaboration with Family AIDS Care and Education Services (FACES), a PEPFAR-funded program that supports over 100 government health facilities in Nyanza Province in HIV prevention, care, and treatment efforts. The program was carried out at a government primary healthcare clinic, which provided all standard primary healthcare services (including antenatal care) as well as HIV care and treatment. 

### 2.2. Program Approach

We followed the six-step process recommended by the WHO for implementing intimate partner violence (IPV) and sexual violence prevention programs [29]. As illustrated in Table 1, we first conducted formative qualitative research, including focus groups with pregnant women and male partners and in-depth interviews with a range of service providers, to learn about pregnant women's experiences of GBV in this rural Kenyan setting [23]. We then convened key stakeholders (representatives from the Ministry of Health, Ministry of Gender and Social Services, nongovernmental organizations (NGOs), faith-based organizations (FBOs), FACES, the police, the judiciary, political leaders, and local community leaders) to review existing models and obtain guidance for developing a GBV program for the rural primary healthcare setting. The insights gained from stakeholders were then used to adapt several existing GBV curricula [33–36] and create a cohesive program package that included (1) building the capacity of health workers, (2) bolstering multisectoral linkages, and (3) enhancing community GBV awareness, with a special focus on reaching men. 

**Figure 1:**
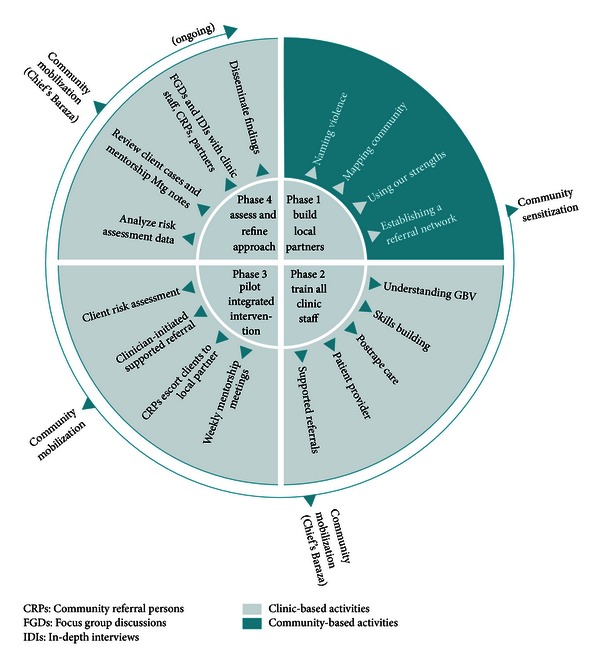
GBV program components.

We conducted a pilot of the program in one community in rural Nyanza Province, during the period November 2010–February 2011. The pilot program was carried out in four phases (see Figure 1) and included both clinic- and community-based activities. No GBV screening or referrals were being conducted at the facility prior to the initiation of this program.

In Phase 1, we built the skills of local community partners (administrative, religious, social, and traditional leaders) to respond to GBV. In a two-day workshop, local partners learned about GBV and effects on health, mapped out their neighborhood, discussed existing (often informal) services that could be supportive of GBV victims, and established a local referral tree.

In Phase 2, we trained all clinic staff (including administrative staff, community volunteers, and lay health workers) through a 40-hour training program. Topics covered in the training included gender and human rights, GBV sensitization, links between GBV and HIV, HIV-related stigma, role of the health sector, privacy and confidentiality, IPV screening tools and techniques, sexual violence and postrape care, supported referral protocols, provider safety and self-care, and communication skills. Two new program tools were developed to facilitate links between the clinic and community. The first was a “risk assessment form,” based on formative research and existing GBV screening tools from South Africa and the United States [37–39]. This form was used to collect data on GBV cases and referrals and served as a guide for healthcare providers on appropriate counseling and referral strategies. The second tool was a specialized “referral tree” containing guidance and contact information for community partners and local resources. Community-based activities during this phase included organization of a local baraza (community meeting held by a local chief) where anti-GBV messages adapted from the Raising Voices curriculum [40] were communicated through posters, speeches, and skits by community groups. 

In Phase 3, the clinic staff began to screen all pregnant women visiting the antenatal care clinic, where women are also tested for HIV and received prevention of mother-to-child transmission (PMTCT) interventions. In lieu of providing all necessary GBV services in the clinic, community volunteers were trained to offer “supported referrals” to services that already existed in the province. Supported referrals are distinct from ordinary referrals because community volunteers offer concrete assistance for reaching referral services in the community or the nearest town, including provision of transport costs, personally escorting women to services, telephoning ahead and offering emotional support. Existing resource persons included medical professionals trained in GBV, community elders, chiefs, counselors, church leaders, police, and a probono lawyer. The community volunteers, called Community Referral Persons (CRPs, 2 women and 3 men), assisted with phoning ahead, escorting women to services, and facilitating reimbursement for transport fare (often a key barrier to accessing support in rural areas). Limited funds were available to support transport costs for clients and CRPs to reach referral agencies in the nearest town, cell phone costs for health workers, and CRPs to communicate with each other and referral agencies and for biweekly meetings of the CRPs led by FACES Coordinators. Throughout this phase, community mobilization events in different parts of the clinic catchment area were conducted by members of the FACES team and the CRPs. 

During Phase 4, we conducted an evaluation of the pilot program by examining risk assessment forms and conducting focus groups and in-depth interviews with community and clinic participants. Evaluation methods and findings are described in detail below.

### 2.3. Evaluation Methods

Due to limited resources and ethical considerations, we conducted a small-scale mixed methods evaluation using anonymous screening from data and qualitative research methods. Although we collected significant data from pregnant women at baseline to inform the design of the program [23], we were not able to trace women who reported violence to collect quantitative data on the outcomes of GBV referrals. In order to avoid putting women at any additional risk due to research procedures [41], we chose to collect stories of the women assisted during confidential in-depth interviews with the community referral persons and other service providers.

*Quantitative.* GBV risk assessment questions routinely asked of antenatal clients during the pilot included the following. (1) If you told your partner that you came here for health services today, would s/he react angrily or negatively? (2) Has your partner or another person close to you: *(a) Pushed, grabbed, slapped, choked hit or kicked you? (b) Threatened to hurt you, your children or someone close to you? (c) Taken away money/resources that you/your children need to survive? (d) Sent you back to your maternal home, (e) Forced you to have sex when you did not want to?* (3) Has your partner tried to get you pregnant when you did not want to be? (4) If you wanted to use a condom or another family planning method, would you be afraid to ask your partner? (5) Are you worried your partner (or another person close to you) will be angry and/or hurt you if s/he finds out you were tested for HIV? (6) Do you feel unsafe returning to your home today? Clients answering “Yes” to any of these questions were considered to be at risk of or experiencing GBV. These anonymous risk assessment forms were completed by the health providers (clinical officers and nurses, both male and female) during antenatal visits at the clinic. The healthcare providers read the questions to the women (in her preferred language) and women orally provided answers that were recorded on the form. Risk assessments took place in a private room in the antenatal clinic, and no other staff or patients were present. Data from these forms (*n* = 134) were examined and simple descriptive statistics were conducted to identify monthly numbers and trends in screening, cases identified, and referral after the initiation of the program activities. 

*Qualitative.* Focus groups with clinic staff and community members were conducted after the program had been active for two months. Participants (2 groups; *n* = 17 participants, both genders) were purposively selected from those who participated in or were affected by the GBV program and included health workers from the clinic, the CRPs, FACES Coordinators, local administrators, church and community leaders, elders, and local nongovernmental organization representatives. Each focus group included a mixture of participants from these target groups. The groups were led by an experienced qualitative researcher in Dholuo or English language (both languages are widely spoken in the district) using a moderator's guide and were audio-recorded after obtaining permission from the participants. Topics included feedback on the overall GBV program approach, the training received, the process of supported referrals; the impact of the program on the clinic, clients, and community; and suggestions for improvement. 

In addition, 5-6 months after the program activities were initiated, we conducted a series of in-depth interviews with key informants (6 women and 19 men) who had been involved in the program. Types of informants included health workers at the clinic (*n* = 5); Ministry of Health, police, and other community, district, and service leaders (*n* = 10); CRPs (*n* = 5); and FACES staff (*n* = 5). These interviews were conducted in English by an experienced qualitative researcher using an in-depth interview guide developed by the research team and were audio-recorded after obtaining permission from the participants. Topics in the interview guide included the participant's role in the community and their overall impression of and role in the program; experiences with GBV risk assessment and the referral process; views on addressing GBV in the community; and facilitators and barriers to program success.

Audio files were transcribed in the original language (Dholuo or English) and then translated to English if necessary by experienced translators based in Kenya. The English transcripts were coded by two researchers in QSR NVivo 9, using a thematic approach to data analysis [42]. An initial coding framework was developed based on several sources: the research questions, data collection themes, and the current literature. Following the development of this initial framework, two authors coded all transcripts according to the identified “broad codes,” which represent wide thematic baskets of ideas [43]. Next, the research team held a series of phone meetings to jointly develop “fine codes” using a grounded theory approach [44]. Two authors then applied the final list of “fine codes” to two separate QSR NVivo databases. A preliminary research report was created by printing out excerpts related to each code, reviewing the text for any divergence of opinion and summarizing the views of participants alongside illustrative quotes. 

*Ethical Considerations.* All participants were taken through an informed consent process and provided verbal informed consent to participate in the research. Ethical approval for this study was obtained from the Kenya Medical Research Institute (KEMRI), the University of California, San Francisco (UCSF), and the University of Alabama at Birmingham (UAB). 

## 3. Results

### 3.1. Screening and Referrals

A total of 134 women were screened in the ANC clinic during the months December 2010 from April 2011. Forty-nine women (37%) reported some type of violence or risk of violence (physical, sexual, and/or psychological). Of the 134 forms, 24 (18%) included a report of physical violence (pushing, grabbing, slapping, choking, hitting, and kicking), 23 (17%) sexual violence (forcing sex), 26 (19%) psychological violence (threatening own safety or children/persons close to you), and 15 (11%) economic violence (forcing out of home or taking away money/resources you or your children need to survive). 

Of those reporting violence, 26 (53%) accepted referrals to support resources in the province (not all women reporting violence wanted to take any action). Support was provided for these women to access these support resources including referrals to community referral persons (23 women), police in the nearest town (4 women) (for filing paper work—the P3 form—to make a formal complaint against the perpetrator of the violence), local government administrators (3 women) (to help with needs for shelter and food), nongovernmental organizations working on women's rights (2 women) (for counseling), a probono lawyer (1 woman) (for those who wanted to pursue legal action), and village elders (1 woman) (for help with communication with the husband and family). 

**Figure 2:**
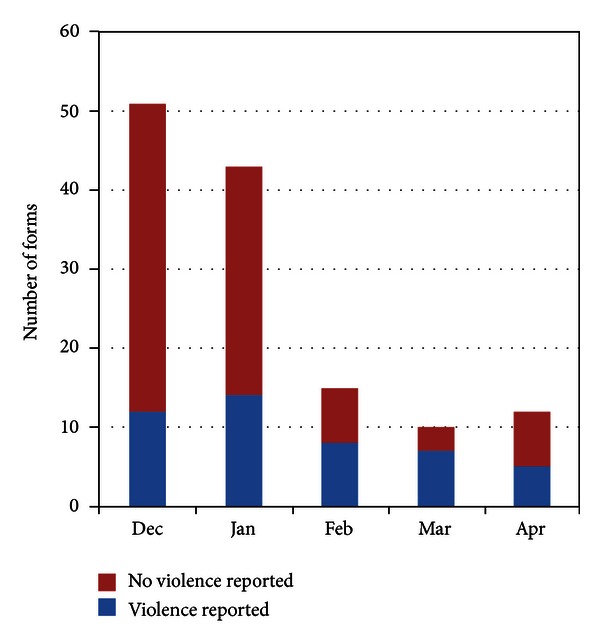
GBV screening by month, December 2010–April 2011.

Examination of numbers of risk assessment forms, including the two months after the initial pilot period ended in February, indicated that the volume of screening in the ANC clinic declined over time, although the number of ANC visitors remained relatively stable (Figure 2). Characteristics of the GBV cases described by the CRPs during in-depth interviews are presented in Table 2. 

### 3.2. Evaluation Focus Groups and In-Depth Interviews with Healthcare Providers and Community Service Providers

The major themes that emerged from the qualitative data analysis included: changes in community GBV awareness, the role of screening at the health facility in facilitating women's access to GBV services, the importance of community collaboration, social risks in the community for persons working to prevent and address GBV, and challenges to program success. 

#### 3.2.1. Theme 1: The Community Gained Awareness of GBV Services and Consequences for Perpetrators

 The participants felt that the GBV program had begun to make a difference in the community. Victims of violence had become aware that there was a way to get help, and perpetrators of violence were learning that there were consequences of partner violence. Several interviews and focus group discussions highlighted the benefit of simply knowing where to receive GBV-related services:

*At least they know there are people somewhere who are out for women. At least there is somewhere where women can file their cases when they are battered, so at least there is a change, they are getting to know that they should do the right thing at the right time, it is not like those days when they use to beat us. (Focus Group #2, Respondent #4) *

One participant explained that the program raised awareness of the consequences of perpetrating violence, creating a disincentive for men to use physical GBV towards their partners:

*It's not the same, because now men fear beating women or doing such violence because they know they may be arrested or there may be steps taken for them if they do that. (In-Depth Interview participant #11)*

#### 3.2.2. Theme 2: Screening for GBV at the Health Facility Opens the Door to Accessing Services

Participants saw benefits to screening at the health facility in rural areas, as women commonly access health clinics but may be less familiar with other available services for GBV. In several focus group discussions, participants explained that GBV screening offered a crucial first step for assisting women with violence:

*This program can be so nice such that at every health facility, any woman walking there can be able to be screened and then any gender-based violence can be identified and then they can be helped… And that can only be generated from the screening and when the screening is not done at that [health] facility level, then you cannot get the other proceedings. (Focus Group #1, Respondent # 5)*

#### 3.2.3. Theme 3: Community Collaboration Can Help GBV Victims in Low-Resource Settings

The involvement of community partners in the GBV program resulted in the ability to find local solutions to help victims, even in this rural setting where there is no battered woman's shelter and limited formal resources for GBV. One participant in a focus group explained how they leveraged space in the chief's home as a way to provide a woman safety while she decided on her next move:

*We accommodated the lady I think for two to three days in the chief's home. He is a simple man, so that is where she stayed, she took a bath she was feeling good… The same lady came back to me and then I found for her shelter with a neighbor,… so I went and talked to that lady and she stayed with her for three days. (Focus Group #1, Respondent #9)*

#### 3.2.4. Theme 4: Those Working to Prevent GBV May Face Negative Community Judgment

Although the health workers and CRPs felt empowered by the training and felt they were doing good in the community, some found the work challenging and were criticized by other community members (especially men) because of their new role:

*So when somebody is saying that women are not supposed to be beaten, that… they should go to somebody and take some action, in the community it is like that person is acting against the will of the community. To the men it is like he is an outcast in the community, an outlaw who is not supposed to be there…. In social places you will hear them saying that he is not a good person because if he is preaching to our ladies and women to take action against us, then it is like he wants to bring a revolution, women are going to overpower us and then we are going to be voiceless. (Focus Group #1, Respondent #8)*

In order to carry out this work more effectively, most participants stressed the need for repeated refresher trainings and sensitization for service providers and local partners (including local administration and police) as well as additional counseling skills for CRPs and health workers.

#### 3.2.5. Theme 5: Despite the Gains, Structural Challenges Remain

Despite their successes, the participants discussed many continuing challenges to addressing GBV in their community. They explained that socioeconomically disempowered women were reluctant to press charges against a violent husband for fear of “breaking the family” and subsequently being left without a home or resources. Some service providers mentioned that they, as well as the women, often preferred to “solve things at home” instead of seeking outside help. Extended family members and village elders (those who had not participated in the local partners meeting) in some cases supported the violent man over the woman. Several participants explained that criminal and legal procedures for reporting GBV cases could not be completed locally but had to be carried out in the nearest town, resulting in difficulties in pressing charges and delays in action:

*Sometimes you get a woman has been beaten by her husband. And when she comes here to report, she reports the matter. Then you start to give her P3 [official violence reporting form]. When she goes home, she's threatened by the family of the husband: “If you go ahead with that case, you are not going to stay. You'll not be here. We will chase you away, if our brother is arrested. (In-Depth Interview participant #20)*

The forms of resistance to this type of program underscore the need to include local partners and community-level education in order to facilitate acceptance of a clinic-based approach to GBV.

## 4. Discussion

The current study suggests that an integrated program in a rural primary healthcare setting in Kenya is acceptable and feasible to both healthcare providers and the surrounding community. Initial assessment suggests that the program has potential to contribute to both primary and secondary prevention of GBV. The program addressed many of the barriers that have been cited as inhibiting the health sector response to GBV, including lack of provider knowledge, insufficient staff training, few existing policies, poor management support for GBV response, and a lack of coordination between the health sector and other services [24, 45]. We found that healthcare providers and community members were motivated to address the issue of GBV and the program was perceived as a positive contribution to their community. 

The program harnessed an important “window of opportunity” among pregnant women attending a rural antenatal clinic. Women in their reproductive years use medical services more frequently than at any other time [7]. This places healthcare providers in a position to build on-going relationships with pregnant women, a prerequisite for identifying and supporting women experiencing violence [46]. 

The 40-hour training program for all clinic staff and the community volunteers seemed to provide the necessary skills for this type of GBV risk assessment and referral work, although periodic refresher trainings would be necessary to address gaps in skills and maintain these tasks over time. As has been found elsewhere [47], data from the risk assessment forms and the focus groups indicated screening in the ANC clinic declined over time after the training. The focus group and in-depth interview findings indicate that clinicians ultimately may have used more of a “case finding” approach, assessing some clients and not others. Case finding, based on the presentation of specific signs or symptoms of abuse, may be preferable for resourced-constrained settings [48]. Larger systems and structural factors, such as regulations requiring forms for reporting violence (P3 form) to be obtained in the nearest town, were difficult to tackle in this small local pilot.

We also found that community collaboration was crucial to the success of the program in this low-resource setting without any shelters or other formal resources for victims of violence. It is recognized that effective GBV referral services need to offer more support than simply handing women a sheet of paper with a list of potential resources [49]. The necessity of engaging the broader community in GBV is increasingly recognized as an essential addition to sub-Saharan African programming [40] and represents an important adaptation from resource-rich settings, who have historically created clinic-only approaches to GBV during pregnancy [50, 51].

Certainly this program did not address all the challenges to primary and secondary prevention of GBV in this setting. A preference among both service providers and clients to “solve things at home” and use “family mediation” approaches to help the couple to live peacefully may be problematic, especially in severe situations when the woman's life is in danger [52]. This finding is consistent with global GBV research showing that women often prefer informal, family-based mechanisms to formal, legal responses [53, 54]. Importantly, as program service providers began to see GBV as a health issue within their scope of work, women also started to change their expectations around the intractable nature of GBV. This is consistent with other findings that shifting service provider attitudes and perceptions are crucial for altering women's acceptance of GBV services [55].

Implementing this pilot GBV program using a six-step process as recommended by the WHO [29] has important strengths. Local stakeholders were involved in the process from the beginning. The design and content of the pilot program was based on formative research in communities where the services were to be instituted and built on successful models for training health workers in GBV that have been used elsewhere [33, 36]. Training of the entire staff of the health facility was important, especially for a resource-constrained setting where patients often rely on nonclinician staff for advice and assistance. Although clinicians conducted the GBV screening, nonclinicians were involved in giving information and support. The involvement of the whole clinic may also increase provider commitment and sustainability of the program [56]. 

However, it should be noted that the pilot was conducted in only one community/clinic, and some special features of this setting may make the strategy less generalizable. Although we built in program evaluation, using anonymous risk assessment form data, focus groups, and in-depth interviews, we were not able to collect data directly from ANC clients on their experiences with the screening and the GBV program. Although we have clinic data on screening and referrals and a wealth of stories from the research participants on the outcome of the GBV cases identified, we were not able to follow women or collect any quantitative data on the outcomes of GBV referrals. Some of these limitations were due to resource constraints, while others had to do with the highly sensitive nature of this topic and the need to avoid putting women at any additional risk due to research procedures [41]. In addition, we did not collect representative quantitative data on the community response to the intervention. Future studies should use ethical and sensitive methods to determine the effects of such GBV screening and referral programs on both community attitudes and outcomes for women. Future research can be guided by the cluster randomized trial design that is currently being used to evaluate community response to *SASA!* Program for preventing violence against women and HIV infection in Uganda [57]. Although the program was clearly of low-cost, as it used existing staff and infrastructure as well as volunteer work, we did not collect specific cost data nor conduct cost or cost-effective analyses. 

## 5. Conclusions

We integrated a GBV program into a rural antenatal clinic that also provides HIV testing and PMTCT services with the participation of the community and primary healthcare workers. This program was found to be acceptable and feasible and has potential to contribute to primary and secondary prevention of GBV. This model may be applicable to address GBV in the multitude of rural communities in Kenya and elsewhere in sub-Saharan Africa, where the majority of the African population live [58]. If this strategy can be scaled up to other primary healthcare clinics, it has potential to impact on the intersecting epidemics of GBV and HIV. 

## Figures and Tables

**Table 1:** Approach for implementing an integrated community-supported clinic-based GBV program.

Implementation steps*	Methods	Key findings
(I) Establish relationships with key partners	Conducted initial discussions with key stakeholders	Local Ministry of Health and FACES leadership were interested in developing methods to address GBV within health services

(II) Define the nature of the problem	(i) FGDs^†^ with pregnant women (*n* = 4 groups) and male partners or relatives of pregnant women (*n* = 4 groups)	(a) Specific types of GBV commonly experienced by women in this setting: beating, forced sex, verbal abuse, denial of reproductive choice, neglect, and being kicked out of their homes
		(b) Triggers for GBV include woman making decisions (e.g., HIV testing) without partner consent, woman failing to perform household duties, man for misallocating money, woman disclosing HIV status, either partner using alcohol, and either partner is suspected of infidelity
	(ii) IDIs^†^ (*n* = 20) with Ministry of Health, Ministry of Gender and Social Services, NGOs, FBOs, health service providers, police, judiciary, and community leaders	(c) Help-seeking behaviors: women were often reluctant to press formal charges, and in many cases preferred to use more informal community and family mechanisms.
		(d) Local resources do exist for GBV, but those that do exist tend to be weak or inefficient and lack linkages to one another
		(e) Primary healthcare workers are trusted service providers, already being accessed by pregnant women in rural areas, and are a potential resource for primary and secondary prevention of GBV.

(III) Identify potentially effective programs	Convened stakeholders to review existing GBV curricula	Relevant portions of GBV curricula for health workers from Kenya, India, South Africa, and Latin America were identified.

(IV) Develop policies and strategies	Designed locally relevant program using formative research and stakeholder input	Components of an effective program, as defined by stakeholders, were as follows:
		(a) building capacity of health workers,
		(b) bolstering multisectoral linkages,
		(c) enhancing community sensitization and awareness (with a special focus on reaching men)

(V) Create an action plan	Established program model	(See Figure 1)

(VI) Evaluate learning	Conducted a mixed-method evaluation using focus groups (*n* = 2 groups) and clinic data on screening and referral	(See Section 3)

*Adapted from the WHO [29].

^†^FGDs: focus group discussions; IDIs: in-depth interviews; NGOs: nongovernmental organizations; FBOs: faith-based organizations.

**Table 2:** GBV cases handled by community referral persons (*n* = 33).

Characteristic	Number of cases
Gender	
Female	30
Male	3
Who referred to CRP	
Clinic	10
Local administrator	8
Village elders	2
Client came directly to CRP	13
Type(s) of violence	
Physical only	12
Sexual only	6
Emotional only	1
Economic only	6
Physical and emotional	1
Physical and economic	5
Physical and sexual	1
Supported referral made to	
District hospital	3
Counselor at women's NGO	9
Probono lawyer	3
Local clinic	6
Local administrator	5
Police	1
Pastor	2

## References

[1] Ellsberg M, Jansen HA, Heise L, Watts CH, Garcia-Moreno C (2008). Intimate partner violence and women’s physical and mental health in the WHO multi-country study on women’s health and domestic violence: an observational study. *The Lancet*.

[2] Romito P, Turan JM, de Marchi M (2005). The impact of current and past interpersonal violence on women’s mental health. *Social Science and Medicine*.

[3] Campbell JC (2002). Health consequences of intimate partner violence. *The Lancet*.

[4] Kenya National Bureau of Statistics (2010). *Kenya Demographic and Health Survey 2008-09*.

[5] Hindin MJ, Kishor S, Ansara DL (2008). *Intimate Partner Violence Among Couples in 10 DHS Countries: Predictors and Health Outcomes*.

[6] Devries KM, Kishor S, Johnson H (2010). Intimate partner violence during pregnancy: analysis of prevalence data from 19 countries. *Reproductive Health Matters*.

[7] Gazmararian JA, Petersen R, Spitz AM, Goodwin MM, Saltzman LE, Marks JS (2000). Violence and reproductive health: current knowledge and future research directions. *Maternal and Child Health Journal*.

[8] Huth-Bocks AC, Levendosky AA, Bogat GA (2002). The effects of domestic violence during pregnancy on maternal and infant health. *Violence and Victims*.

[9] Rodrigues T, Rocha L, Barros H (2008). Physical abuse during pregnancy and preterm delivery. *The American Journal of Obstetrics and Gynecology*.

[10] Kaye DK, Mirembe FM, Bantebya G, Johansson A, Ekstrom AM (2006). Domestic violence during pregnancy and risk of low birthweight and maternal complications: a prospective cohort study at Mulago Hospital, Uganda. *Tropical Medicine and International Health*.

[11] Boy A, Salihu HM (2004). Intimate partner violence and birth outcomes: a systematic review. *International Journal of Fertility and Women’s Medicine*.

[12] Rico E, Fenn B, Abramsky T, Watts C (2011). Associations between maternal experiences of intimate partner violence and child nutrition and mortality: findings from demographic and health surveys in Egypt, Honduras, Kenya, Malawi and Rwanda. *Journal of Epidemiology and Community Health*.

[13] Jewkes RK, Dunkle K, Nduna M, Shai N (2010). Intimate partner violence, relationship power inequity, and incidence of HIV infection in young women in South Africa: a cohort study. *The Lancet*.

[14] Decker MR, Seage GR, Hemenway D (2009). Intimate partner violence functions as both a risk marker and risk factor for women’s HIV infection: findings from indian husband-wife dyads. *Journal of Acquired Immune Deficiency Syndromes*.

[15] Fonck K, Els L, Kidula N, Ndinya-Achola J, Temmerman M (2005). Increased risk of HIV in women experiencing physical partner violence in Nairobi, Kenya. *AIDS and Behavior*.

[16] Gray RH, Li X, Kigozi G (2005). Increased risk of incident HIV during pregnancy in Rakai, Uganda: a prospective study. *The Lancet*.

[17] Mugo NR, Heffron R, Donnell D (2011). Increased risk of HIV-1 transmission in pregnancy: a prospective study among African HIV-1-serodiscordant couples. *AIDS*.

[18] Turan JM, Bukusi EA, Onono M, Holzemer WL, Miller S, Cohen CR (2011). HIV/AIDS stigma and refusal of HIV testing among pregnant women in rural Kenya: results from the MAMAS study. *AIDS and Behavior*.

[19] Hatcher AM, Turan JM, Leslie HH (2012). Predictors of linkage to care following community-based HIV counseling and testing in rural Kenya. *AIDS and Behavior*.

[20] Kenya Ministry of Medical Services (2011). *Kenya Service Provision Assessment Survey 2010*.

[21] Turan JM, Hatcher AH, Medema-Wijnveen J (2012). The role of HIV-related stigma in utilization of skilled childbirth services in rural Kenya: a prospective mixed-methods study. *PLOS Medicine*.

[22] Bond V, Chase E, Aggleton P (2002). Stigma, HIV/AIDS and prevention of mother-to-child transmission in Zambia. *Evaluation and Program Planning*.

[23] Hatcher AM, Romito P, Odero M, Bukusi EA, Onono M, Turan JM (2013). Social context and drivers of intimate partner violence in rural Kenya: implications for the health of pregnant women. *Culture, Health and Sexuality*.

[24] Colombini M, Mayhew S, Watts C (2008). Health-sector responses to intimate partner violence in low- and middle-income settings: a review of current models, challenges and opportunities. *Bulletin of the World Health Organization*.

[25] O’Reilly R, Beale B, Gillies D (2010). Screening and intervention for domestic violence during pregnancy care: a systematic review. *Trauma, Violence, and Abuse*.

[26] O’Campo P, Kirst M, Tsamis C, Chambers C, Ahmad F (2011). Implementing successful intimate partner violence screening programs in health care settings: evidence generated from a realist-informed systematic review. *Social Science and Medicine*.

[27] Ranney ML, Rennert-May E, Spitzer R, Chitai MA, Mamlin SE, Mabeya H (2011). A novel ED-based sexual assault centre in western Kenya: description of patients and analysis of treatment patterns. *Emergency Medicine Journal*.

[28] Laisser RM, Nystrom L, Lindmark G, Lugina HI, Emmelin M (2011). Screening of women for intimate partner violence: a pilot intervention at an outpatient department in Tanzania. *Global Health Action*.

[29] World Health Organization (2010). *Preventing Intimate Partner and Sexual Violence Against Women: Taking Action and Generating Evidence*.

[30] NASCOP (2009). *Kenya AIDS Indicator Survey 2007*.

[31] National AIDS and STI Control Programme (2008). Kenya AIDS Indicator Survey, KAIS, 2007. *Preliminary Report*.

[32] Central Bureau of Statistics (CBS) [Kenya] (2010). *Kenya Demographic & Health Survey 2008-2009*.

[33] Jewkes R, Christofides N, Mooideen C, Ngubeni R (2007). *Vezimfihlo! A Training Manual for Addressing Gender-Based Violence in VCT*.

[34] PATH (2003). *Ensuring Privacy and Confidentiality in Reproductive Health Services: A Training Module and Guide for Service Providers*.

[35] Fisher D, Lang K, Wheaton J (2010). *Training Professionals in the Primary Prevention of Sexual and Intimate Partner Violence: A Planning Guide*.

[36] Kidd R, Prasad N, Tajuddin M, Avula J, Ginni R, Duvvury N (2007). *Reducing HIV Stigma and Gender Based Violence: Toolkit for Health Care Providers in India*.

[37] Christofides N, Jewkes R (2010). Acceptability of universal screening for intimate partner violence in voluntary HIV testing and counseling services in South Africa and service implications. *AIDS Care*.

[38] Coker AL, Flerx VC, Smith PH, Whitaker DJ, Fadden MK, Williams M (2007). Partner violence screening in rural health care clinics. *The American Journal of Public Health*.

[39] Kiely M, El-Mohandes AA, El-Khorazaty MN, Gantz MG (2010). An integrated intervention to reduce intimate partner violence in pregnancy: a randomized controlled trial. *Obstetrics and Gynecology*.

[40] Raising Voices (2009). *SASA! An Activist Kit for Preventing Violence Against Women and HIV*.

[41] Ellsberg M, Heise L (2005). *Researching Violence Against Women: A Practical Guide for Researchers and Activists*.

[42] Braun V, Clarke V (2006). Using thematic analysis in psychology. *Qualitative Research in Psychology*.

[43] Miles M, Huberman A (1994). *Qualitative Data Analysis: An Expanded Sourcebook*.

[44] Hutchison AJ, Johnston LH, Breckon JD (2010). Using QSR-NVivo to facilitate the development of a grounded theory project: an account of a worked example. *International Journal of Social Research Methodology*.

[45] D’Avolio DA (2011). System issues: challenges to intimate partner violence screening and intervention. *Clinical Nursing Research*.

[46] Anderson BA, Marshak HH, Hebbeler DL (2002). Identifying intimate partner violence at entry to prenatal care: clustering routine clinical information. *Journal of Midwifery and Women’s Health*.

[47] McLeer SV, Anwar R (1989). A study of battered women presenting in an emergency department. *The American Journal of Public Health*.

[48] Joyner K, Mash RJ (2011). The value of intervening for intimate partner violence in South African primary care: project evaluation. *BMJ Open*.

[49] Krasnoff M, Moscati R (2002). Domestic violence screening and referral can be effective. *Annals of Emergency Medicine*.

[50] Parker B, McFarlane J, Soeken K, Silva C, Reel S (1999). Testing an intervention to prevent further abuse to pregnant women. *Research Nursing Health*.

[51] Tiwari A, Leung WC, Leung TW, Humphreys J, Parker B, Ho PC (2005). A randomised controlled trial of empowerment training for Chinese abused pregnant women in Hong Kong. *BJOG: An International Journal of Obstetrics and Gynaecology*.

[52] Romito P (2008). *A Deafening Silence: Hidden Violence Against Women and Children*.

[53] Fanslow JL, Robinson EM (2010). Help-seeking behaviors and reasons for help seeking reported by a representative sample of women victims of intimate partner violence in New Zealand. *Journal of Interpersonal Violence*.

[54] Kiss L, d'Oliveira AF, Zimmerman C, Heise L, Schraiber LB, Watts C (2012). Brazilian policy responses to violence against women: government strategy and the help-seeking behaviors of women who experience violence. *Health and Human Rights*.

[55] Christofides NJ, Muirhead D, Jewkes RK, Penn-Kekana L, Conco DN (2006). Women’s experiences of, and preferences for, services after rape in South Africa: interview study. *The British Medical Journal*.

[56] Rogow D (2006). *Living Up to Their Name: Profamilia Takes on Gender-Based Violence*.

[57] Abramsky T, Devries K, Kiss L (2012). A community mobilisation intervention to prevent violence against women and reduce HIV/AIDS risk in Kampala, Uganda (the SASA! study): study protocol for a cluster randomised controlled trial. *Trials*.

[58] International Fund for Agricultural Development (IFAD) (2010). *Rural Poverty Report 2011*.

